# INITIAL FINDINGS ON THE BENEFITS OF THE TOVERTAFEL: REDUCING BEHAVIORS IN PERSONS WITH DEMENTIA

**DOI:** 10.1093/geroni/igad104.2653

**Published:** 2023-12-21

**Authors:** Victoria Steiner, Jennifer Perion, Meggan Hartzog, Safa Ibrahim, Alexa Lopez, Beanna Martinez, Barbara Saltzman, Jennifer Kinney

**Affiliations:** University of Toledo, Toledo, Ohio, United States; The University of Tennessee, Knoxville, Knoxville, Tennessee, United States; University of Toledo, Toledo, Ohio, United States; University of Toledo, Toledo, Ohio, United States; University of Toledo, Toledo, Ohio, United States; University of Toledo, Toledo, Ohio, United States; University of Toledo, Toledo, Ohio, United States; Miami University, Oxford, Ohio, United States

## Abstract

Technologies have been developed to reduce behaviors in persons with dementia by addressing unmet needs through activities that stimulate multiple senses. The Tovertafel (“magic table”) is a novel device from the Netherlands that enables purposeful play using interactive games designed to promote social, physical, and cognitive engagement in people with dementia. This study is investigating whether an intervention using the Tovertafel improves quality of life for residents with moderate to severe dementia living in long-term care communities in the Midwest. Twelve white women with an average BIM score of 6 completed the study at the initial facility. The 4-week intervention consisted of 30-minute game sessions with 2 to 6 individuals each weekday. Participants attended 2.5 sessions per week on average and displayed increased engagement during the games per facilitator reports. Direct care staff completed the Revised Memory and Behavior Problems Checklist–Nursing Home and the Apathy Evaluation Scale–10 for all participants. Data collected eight times over six consecutive weeks during the control (2 weeks) and intervention (4 weeks) periods were analyzed. After checking assumptions, paired-sample t-tests were conducted that indicated significantly fewer memory-related behaviors (p=.047) (e.g., repetitive behaviors and questions) and instances of apathy (p=.027), during the intervention period than the control. Despite reductions in depressive and disruptive behaviors, no statistically significant differences were found. Initial findings suggest that the Tovertafel could offer innovative ways to provide activities to people with moderate to severe dementia in long-term care settings, increase their quality of life, and improve interactions with staff.

